# Nanomaterials in Animal Husbandry: Research and Prospects

**DOI:** 10.3389/fgene.2022.915911

**Published:** 2022-06-21

**Authors:** Kun Wang, Xubin Lu, Yi Lu, Jiacheng Wang, Qinyue Lu, Xiang Cao, Yi Yang, Zhangping Yang

**Affiliations:** ^1^ College of Animal Science and Technology, Yangzhou University, Yangzhou, China; ^2^ Joint International Research Laboratory of Agriculture & Agri Product Safety, Ministry of Education, Yangzhou University, Yangzhou, China; ^3^ Jiangsu Co-innovation Center for Prevention and Control of Important Animal Infectious Diseases and Zoonoses; College of Veterinary Medicine, Yangzhou University, Yangzhou, China; ^4^ College of Medical, Yangzhou University, Yangzhou, China

**Keywords:** nanomaterial, nanoparticles, anti-inflammatory, antitumor, animal medicine

## Abstract

Anti-inflammatory, antiviral, and anti-cancer treatments are potential applications of nanomaterials in biology. To explore the latest discoveries in nanotechnology, we reviewed the published literature, focusing on co-assembled nanoparticles for anti-inflammatory and anti-tumor properties, and their applications in animal husbandry. The results show that nanoparticles have significant anti-inflammation and anti-tumor effects, demonstrating broad application prospects in animal breeding. Furthermore, pooled evidence suggests that the mechanism is to have a positive impact on inflammation and tumors through the specific drug loading by indirectly or directly targeting the disease sites. Because the precise regulatory mechanism remains unclear, most studies have focused on regulating particular sites or even specific genes in the nucleus by targeting functional co-assembled nanoparticles. Hence, despite the intriguing scenarios for nanotechnology in farmed animals, most results cannot yet be translated into field applications. Overall, nanomaterials outperformed similar materials in terms of anti-inflammatory and anti-tumor. Nanotechnology also has promising applications in animal husbandry and veterinary care, and its application and development in animal husbandry remain an exciting area of research.

## 1 Introduction

The term “nanotechnology” was coined by Nobel Laureate Richard P. Feynman in his speech “There’s Plenty of Room at the Bottom” (Ijaz et al., 2020) in 1959. Nanomaterials have unique physical, chemical, and biological properties compared with non-nanomaterial counterparts. Due to small physical size, molecules, biologically more active and soluble, have more stable structure and are less impacted by oxidative inactivation and other underlying factors (Swain et al., 2015). Made by nanotechnology, Nanoparticles (NPs) come in a variety of shapes, including spheres, wires, and stars, and are widely used in medicine and animal husbandry because of unique qualities.

Nanobiotechnology is the application of nanotechnology to biological sciences (Swain et al., 2015). Since the advent of “nanotechnology”, its field has witnessed a sequence of breakthroughs. A nanoparticle is originally defined as a particle with the size of 1–100 nanometres (nm) (Laurent et al., 2008), whereas in the biomedical field, it is now considered to be between 1–1,000 nm. In the field of medical research, the advancement of nanomaterials has boosted the prevalent use of nanocarriers, particularly in the field of medication and gene delivery. In addition, application of nanomaterials in nanomedicine can treat medical problems and diseases more rapidly and effectively. Consequently, researchers use the knowledge of these processes to develop new therapy options and conceptual approaches for a variety of medical issues (Dobrovolskaia et al., 2016). Nano-targeted drug delivery systems, for example, have the potential to improve drug solubility, bioavailability, activity, and safety. In preclinical models, nanoformulations can slow drug release rate, prolong the blood circulation time of drugs, and reduce the needed dose, improving its pharmacodynamic effect compared to regular pharmaceutical formulations (Halwani, 2022).

Research has demonstrated that drug development schemes and treatment methods based on nanomaterial technology show distinctive superiority in anti-inflammatory and anti-tumor pursuits. This is because drug-loaded NPs are designed to be targeted, as opposed to traditional drug therapy’s “indiscriminate” therapeutic strategy. It is a treatment that can target and destroy certain types of sick tissues while retaining healthy cells. NPs with specific properties are designed for targeted therapy to transport therapeutic agents to tumor sites and release them under controlled conditions (Sohrabi Kashani and Packirisamy, 2021). By securely delivering therapeutic medications to damaged areas or specific cells, these technologies may be able to overcome the constraints of previous methods and improve the therapeutic effect (Jurj et al., 2017; Mu et al., 2020).

In this review, we focus on the properties of various nanomaterials and their clinical and preclinical studies in anti-inflammatory and antiviral research. In addition, research directions of future nanotechnology and possibilities in animal husbandry and veterinary medicine are also presented. This review purports to aid researchers in better understanding the mechanism of nanomaterials in anti-inflammatory and antiviral aspects, provide experimental ideas and theoretical foundations for nanotechnology development, and assist researchers in exploring the application of nanomaterials in animal husbandry and veterinary medicine.

## 2 Origin and Synthesis of NPs

NPs are composed of three layers: (A) a surface layer, which can be functionalized with a variety of small molecules, metal ions, surfactants, and polymers; (B) an outer shell layer; and (C) a core moiety, which refers to the NPs themselves (Shin et al., 2016). With the advance of nanotechnology and creation of new NPs, the known structure of NPs is evolving, particularly via co-assembly and bio NPs (Noman et al., 2022).

### 2.1 Classification of NPs

**FIGURE 1:**
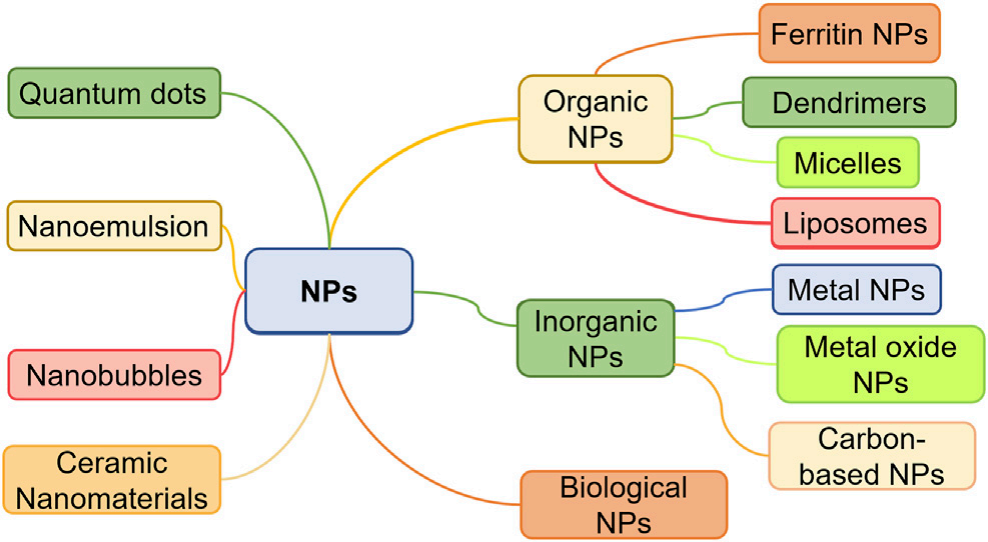
Nanoparticle classification.

The morphology, size, and form of NPs are usually used to classify them. There are three categories of scale-based nanomaterials, that is one-dimensional (1D), two-dimensional (2D), and three-dimensional (3D) (Ijaz et al., 2020). Moreover, material standards are frequently used to classify items, such as polymeric NPs, liposome-based NPs, natural NPs, inorganic NPs (Figure 1).

### 2.2 Synthesis of NPs

According to the synthesis orientation, nanoparticle synthesis is classified as bottom-up or top-down (Camargos and Rezende, 2021). These methods are further subdivided into several sub-categories in the light of operation, reaction conditions, and procedure. The top-down synthesis uses destructive methods to break down materials from larger molecules into smaller ones, which are then turned into appropriate NPs. This process is also known as the Building Up Method since the synthesized NPs are made from relatively simple components. Examples of top-down synthesis include mechanical/ball-milling procedures, thermal decomposition methods, lithography methods, laser ablation, and physical vapor deposition. By comparison, the bottom-up method is known as the construction method. NPs created by this process are relatively simple materials. Examples of this synthesis include chemical vapor deposition, sol-gel technique, Pyrolysis, spinning, and biological synthesis.

Nanoparticle synthesis technology is evolving rapidly, and these days it can be divided into three types: biological technique, physical technique, and chemical technique, among which the biological one has increasingly become the optimum method because of its simplicity, non-toxicity, and economic benefits. Different synthesis methods endow NPs with different characteristics, thus the appropriate synthesis method must be chosen based on the expected results. If you wish to make NPs with magnetic characteristics, for example, you can employ synthesis processes, including co-precipitation, microemulsion, thermal breakdown, and flame spray (Wu et al., 2008). Furthermore, hazardous substances, frequently utilized in the chemical and physical synthesis of NPs, can lead to environmental problems. As a result, less harmful biological approaches are often the preferred option for preparing NPs.

## 3 Anti-inflammatory Effects of NPs

### 3.1 Anti-inflammatory Properties of NPs

Inflammation is the defense response of organisms against infections or tissue damage caused by invading microorganisms or other reasons (Kolaczkowska and Kubes, 2013). Excessive inflammation can lead to autoimmune or inflammatory disorders, such as acute respiratory distress syndrome (ARDS), sepsis, stroke, and rheumatoid arthritis. Researchers have developed good anti-inflammatory medicines and anti-cytokine therapies to minimize cytokine storms and suppress inflammation, but non-targeted delivery and immune system damage can result in adverse effects (Wang et al., 2014). One of the reasons why most antioxidants are ineffective in inflammatory disease clinical trials is the restricted ability of a single antioxidant that scavenges reactive oxygen species (ROS). Hence, researchers need to develop other anti-oxidative stress strategies (Ingle et al., 2014). Selective targeting, precise intracellular delivery, and induction of immune cell apoptosis are techniques for reducing inflammatory reactions.

Traditional approaches for detecting and treating inflammation have been extended by the rapid development of nanotechnology. NPs have an increased permeability and retention effect (EPR) that allows them to preferentially aggregate in tumor tissues (Zhi et al., 2020). For anti-inflammatory and antioxidant activities, there is growing evidence that nanoparticle-based targeted techniques are promising. Sugar-based amphiphilic NPs, for example, can effectively treat atherosclerosis by competitively limiting oxidized lipids absorption of macrophage scavenger receptor (Rai et al., 2016). Another example is that drug-induced intestinal inflammation and nonalcoholic steatohepatitis could be efficiently treated with nanotherapies made from Tempol-containing amphiphilic copolymers (Botequim et al., 2012; Akhtar et al., 2019). Furthermore, DSPE-PEG2K-modified cerium oxide nanozymes (CeNZs) can remove ROS such as H_2_O_2_, O^2−^, and OH in injured hepatocytes and reduce oxidative stress in a damaged liver. Oxygen can reduce the number of pro-inflammatory macrophages, thus preventing inflammation that leads to hepatocyte necrosis (Habiba et al., 2016).

Pneumonia caused by COVID-19 poses a major hazard to people’s health and life, particularly to the elderly and those with lung disorders. To control it, useful medications must be developed. Nanomaterial will be a viable treatment to save more lives since nanomaterial makes it easier to create simple, quick, and low-cost diagnostic assays for SARS-CoV-2 and associated biomarkers (Zeng et al., 2021). Studies have shown that nanomaterial can effectively deliver viral antigens to antigen-presenting cells or serve as adjuvants in the host, so as to produce vaccine rapidly (Zeng et al., 2022). And treatments based on nanomaterials may also decrease SARS-CoV-2 multiplication and reduce inflammation (Phuna et al., 2022).

### 3.2 Anti-inflammatory Mechanism of NPs

Studies have shown that NPs tend to accumulate in mitochondria and interfere with cellular antioxidant defense mechanisms to achieve anti-inflammatory effects (El-Sayed and Kamel, 2020). Some researchers have found that cerium oxide NPs can upregulate the expression of Nrf-2 in cells, activate the Nrf-2/Keap1 pathway, and significantly upregulate the expression of antioxidant genes (Nqo1, Gpx1, and HO-1). They can also suppress the expression of antioxidant genes (Nox2 and Cyp2e1). CeNZs can scavenge ROS and generate oxygen to decrease inflammation by mimicking functions of catalase and superoxide dismutase (Li et al., 2020). In addition, NPs can achieve targeting to reduce the required drug dosage through the overexpression of activated neutrophil surface characteristic receptors, pH-triggered drug release through pH-responsive bonds, and the high apoptosis-inducing ability of the loaded drugs. NPs in the range of 100–200 nm have been shown to extravasate through vascular fenestrations of tumors and escape filtration of liver and spleen. This means NPs are not cleared by the kidney and reticuloendothelial system, allowing the drug to be effectively delivered to the target site with vascular permeability (Blanco et al., 2015). Thus, NPs can play a dual regulatory role in the treatment of inflammation, scavenging ROS in damaged cells, reducing oxidative stress at the damaged site, and producing oxygen to lower the number of pro-inflammatory macrophages, thereby preventing further inflammatory responses caused by inflammation and apoptosis.

In general, the majority of relevant research has confirmed the obvious benefits of NPs in anti-inflammatory, which are based on anti-inflammatory medication treatment and the advantages of nanomaterials. More specific pathways can be found to improve anti-inflammatory effects of NPs after in-depth studies of NPs.

## 4 Nanoparticle Antitumor Properties

### 4.1 Antitumor Effect of NPs

The anti-tumor effect of NPs is often obtained through the therapeutic effect of medicine. Improved anti-tumor effects can be achieved by combining features of NPs such as EPR effect, biosafety, and biocompatibility, among others. Researchers, for example, have coupled mesoporous silica NPs (MSN) with doxorubicin (DOX), a potent anti-cancer medication. To encapsulate DOX into MSNs, a specially designed wrapping DNA was used as the bio-gate of telomerase-response (Ma et al., 2021). In the presence of highly activated telomerase in tumor cells, the surrounding DNA is extended, preventing the drug from working in normal cells.

**FIGURE 2:**
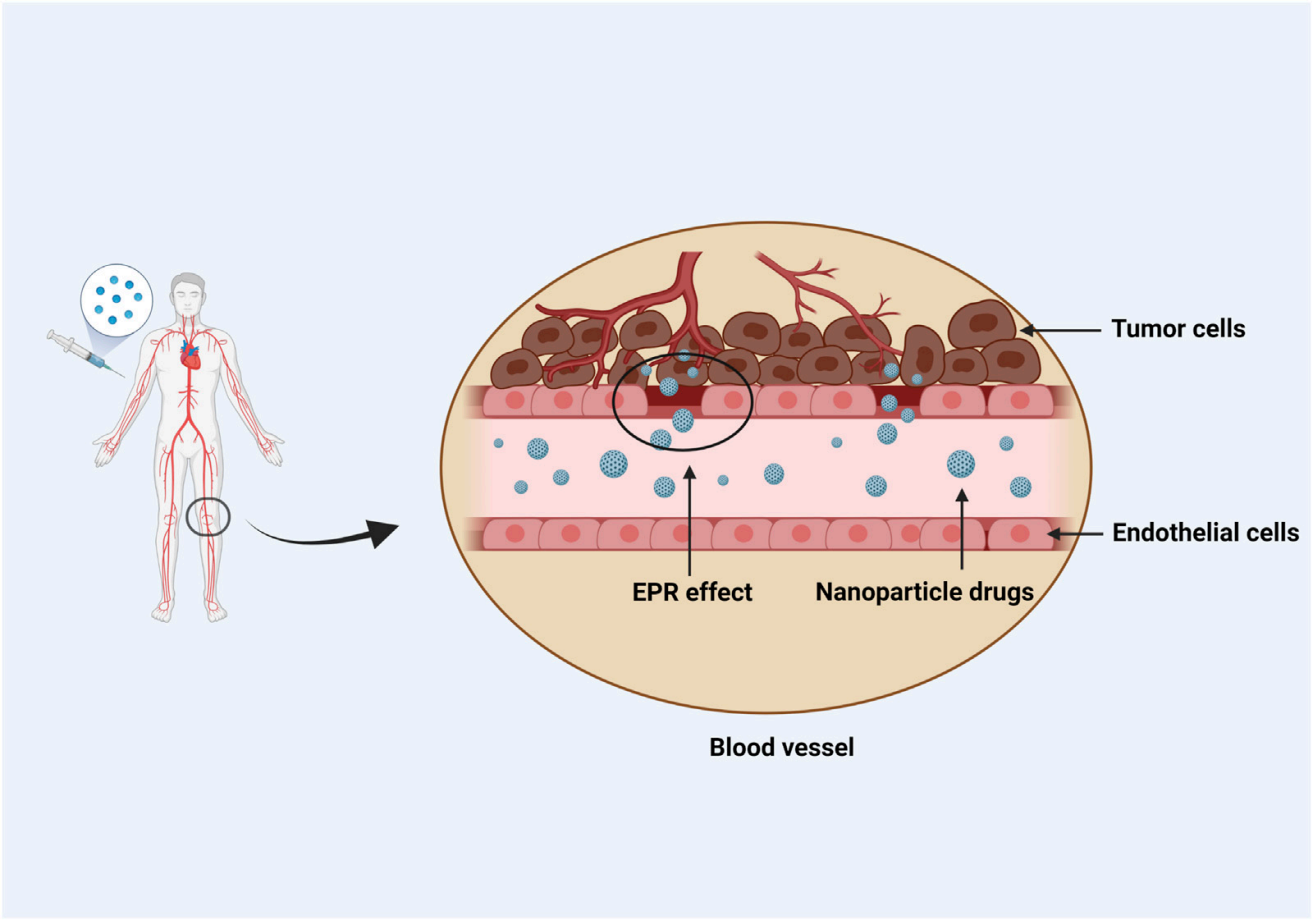
NPs passively target the local environment of tissues. To enhance uptake and accumulation in tumor tissues and inflammatory regions, passive targeting depends on cell-specific functions or local conditions particular to the target tissue. Blood vessels abound in tumor tissues, which have large vascular wall gaps and poor structural integrity. Due to the EPR effect, nanomedicines with a diameter of 10–100 nm can be concentrated in tumor tissues. On the contrary, because the microvascular endothelial space in healthy tissue is dense and structurally intact, macromolecules and lipid particles are difficult to penetrate the blood vessel wall, resulting in a decrease in nanomedicine distribution in normal tissues and passive targeting of nanomedicine to tumor tissue.

NPs can not only be employed as anticancer drug carriers, but also improve solubility of standard drug formulations and minimize side effects and immunotoxicity. For example, the cancer drug paclitaxel (PTX) can cause allergic reactions in some people, but these reactions can be reduced to some extent when PTX is combined with NPs to produce nano albumin formulations (nab-PTX) (Swain et al., 2015). Micellar PTX, a novel nontoxic nanomedicine, can improve the overall response rate and biological observation response rate of malignant mast cell tumors in dogs as compared with lomustine. Nanotubes with particular structures can enter cells through endocytosis and selectively kill malignant tumor tissues. At the same time, they are easily absorbed and transported in the cell membrane due to their needle-like features (Jurj et al., 2017). Drugs could be targeted to tumors preferentially via “active targeting,” which involves utilization of a peptide or antibody that binds to a protein selectively expressed on cancer cells. To enhance the uptake and accumulation in tumor tissues and inflammatory regions, drugs should “passively target” cell-specific functions or local surroundings (Figure 2).

Nanomaterial-based antitumor drug delivery systems provide the following advantages: 1) high payload drug combinations; 2) improved pharmacokinetics and bioavailability of included medicines; 3) surface functionalization. Nanodrug delivery systems based on NSMs with self-assembly capabilities have remarkable biological activity. Examples include dehydrotrametenolic acid NPs for oral drug delivery, NSM sterols for antitumor photodynamic therapy while enhancing biosecurity, NSM gel for *in situ* injection therapy treatment while reducing the body inflammatory response, and ursolic acid drug-loaded NPs for synergistic antitumor therapy while reducing side effects of chemotherapy drugs (Cheng et al., 2019; Yang et al., 2019; Wang et al., 2020a).

Alternative delivery strategies for functionalization of miRNA and siRNA have received much attention recently. In multicellular animals, including mammals, miRNAs play a crucial role in biochemical pathways (Tian et al., 2022). When miRNAs are dysregulated, unbalanced gene expression is related to the deregulation of important cellular processes (Kim et al., 2011). As therapeutic agents, various types of miRNAs have been placed on the surface of NPs. Similarly, there are many potential problems in the systemic delivery of siRNA in tumor therapy, including interaction with specific gene targets, difficulty in administration, low circulation stability, increased cellular uptake, the difficulty of distribution monitoring, and adverse therapeutic effects (Jurj et al., 2017).

### 4.2 Antitumor Mechanism of NPs

The non-destructive diagnosis and tailored therapy of tumors *in vivo* are based on the specific enrichment of nanomaterials in tumor tissue. This is primarily accomplished through two mechanisms: passive targeting, which employs the EPR effect, and active targeting, which involves loading nanomaterials with tumor marker ligands. In other words, passive targeting promotes accumulation of nanocarriers in solid tumors, active targeting provides an additional layer of tunable control and widens the therapeutic window. However, some researchers believe that active targeting does not promote or prevent the enrichment of nanomaterials in tumors (Bhattacharyya et al., 2015; Costa et al., 2019). In addition, the cytotoxicity of active targeting NPs was significantly greater than that of non-targeting control groups in multiple cancer cell lines. As a result, more research is needed to uncover the mechanisms of NPs so that their application potential can be fully realized.

**FIGURE 3:**
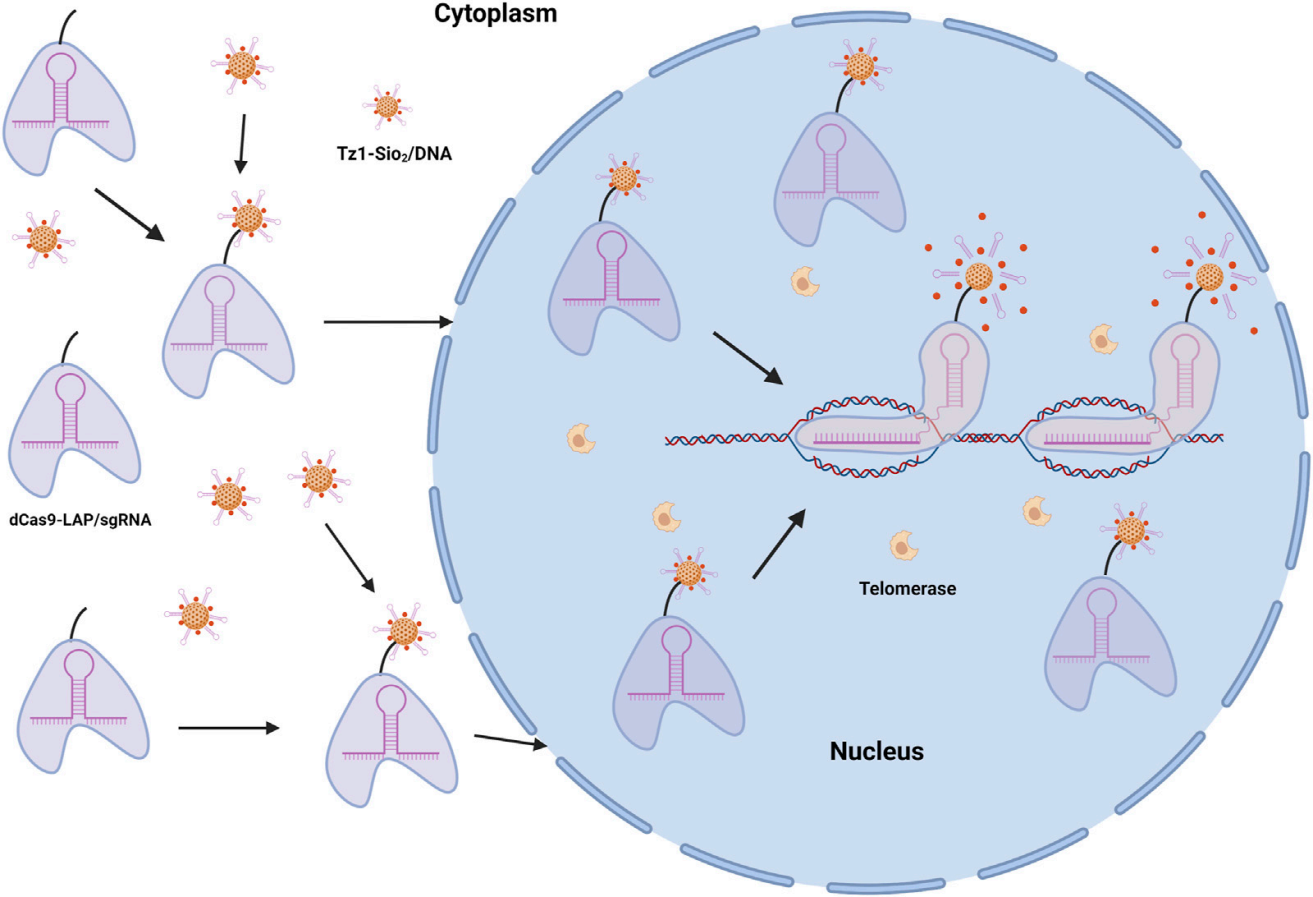
dCas9-MSN/DOX/DNA drugs target cancer cell nucleus and release. DOX, the primary anticancer agent, is encapsulated in MSNs to produce the telomerase-responsive biogate (MSNs/DOX/DNA). In the presence of telomerase, which is extremely active in tumor cells but inert in normal healthy cells, the time of wrapping DNA can be prolonged.

Nanodrug systems are an effective anticancer drug delivery technique that reduces toxic side effects while increasing therapeutic efficacy (Li et al., 2018; Wang et al., 2018). Nanodrug delivery technique has the potential of efficient anticancer medication delivery. Current nanosystems are frequently found in the cytoplasm and mediate uncontrolled release of medication into the cytoplasm, whereas many anticancer treatments are more effective in the nucleus. As a result, it is necessary to further investigate the targeting strategy of nanodrug carriers for the nucleus. For nuclear targeting and intelligent release of anticancer medication, a CRISPR-dCas9-guided telomerase-responsive nanosystem has been developed (Ma et al., 2021). This design precisely locates nanocarriers in the nuclear genome, delivers anti-tumor medications through the specified nuclear-targeting mechanism, eliminates the interference of biological barrier and low non-specific diffusion, resulting in a dramatic increase of anti-cancer activity (Figure 3). As a result, this innovative nano targeting technology is expected to become a platform for high-precision drug delivery and controlled release of anticancer medicines, thereby improving cancer therapeutic efficiency.

Magnetic thermotherapy (MTT), a unique anti-tumor physical therapy, has piqued the interest of researchers. Using the thermal effect of magnetic NPs under the action of alternating magnetic fields, as well as the characteristics of tumor cells that are less thermally tolerant than normal cells, it selectively kills tumor cells by injecting magnetic NPs into tumor sites before applying the alternating magnetic fields (Farzin et al., 2020). Some researchers initially proposed magnetothermodynamic therapy (MTD), which overcomes the limitations of standard MTT by combining magneto-induced thermal actions of nanoiron oxide with its ROS-related immunological effects to successfully prevent the growth of tumor (Liu et al., 2020). MTD facilitates the growth of traditional MTT and addresses the shortcoming that MTT solely depends on the thermal effect. MTD not only dramatically improves anti-tumor efficacy by combining the immunological impact caused by ROS, but also presents a new notion for precise and efficient nanomagnetic therapy.

Compared with traditional antitumor medications, the targeting and EPR effect of nanomedicines are more effective in treatment, safer in treatment, and less harmful to living organisms. On the ground that their functionality has not been fully developed, further research is needed. In addition, due to the imperfection of the targeted regulatory approaches, further research into these findings is recommended to improve the intelligent release of NPs in tumor tissues and provide a more accurate theoretical foundation for the treatment of malignancies with nanomedicines.

## 5 Functional Identification and Tools of NPs

Biodistribution, biocompatibility, biodegradability, and systemic clearance are general problems of employing NPs in targeted therapy (Sun et al., 2014). The physical and chemical properties of NPs have a significant impact on their interaction behavior. To develop safe NPs and govern its targeting, it is required to thoroughly understand the relationship and their attributes of NP cellular absorption, nanotoxicity, and intracellular distribution (Yameen et al., 2014; Behzadi et al., 2017). Herein, the analysis methods of NPs include dynamic light scattering, scanning electron microscope, energy dispersive spectroscopy, UV-Vis spectroscopy, X-ray diffraction, Fourier transform infrared spectroscopy, surface-enhanced residual spectroscopy, atomic force microscopy, high angle annular dark field, atomic absorption spectroscopy, and ray photoelectron spectroscopy (Ijaz et al., 2020).

### 5.1 Cellular Uptake of NPs

**FIGURE 4:**
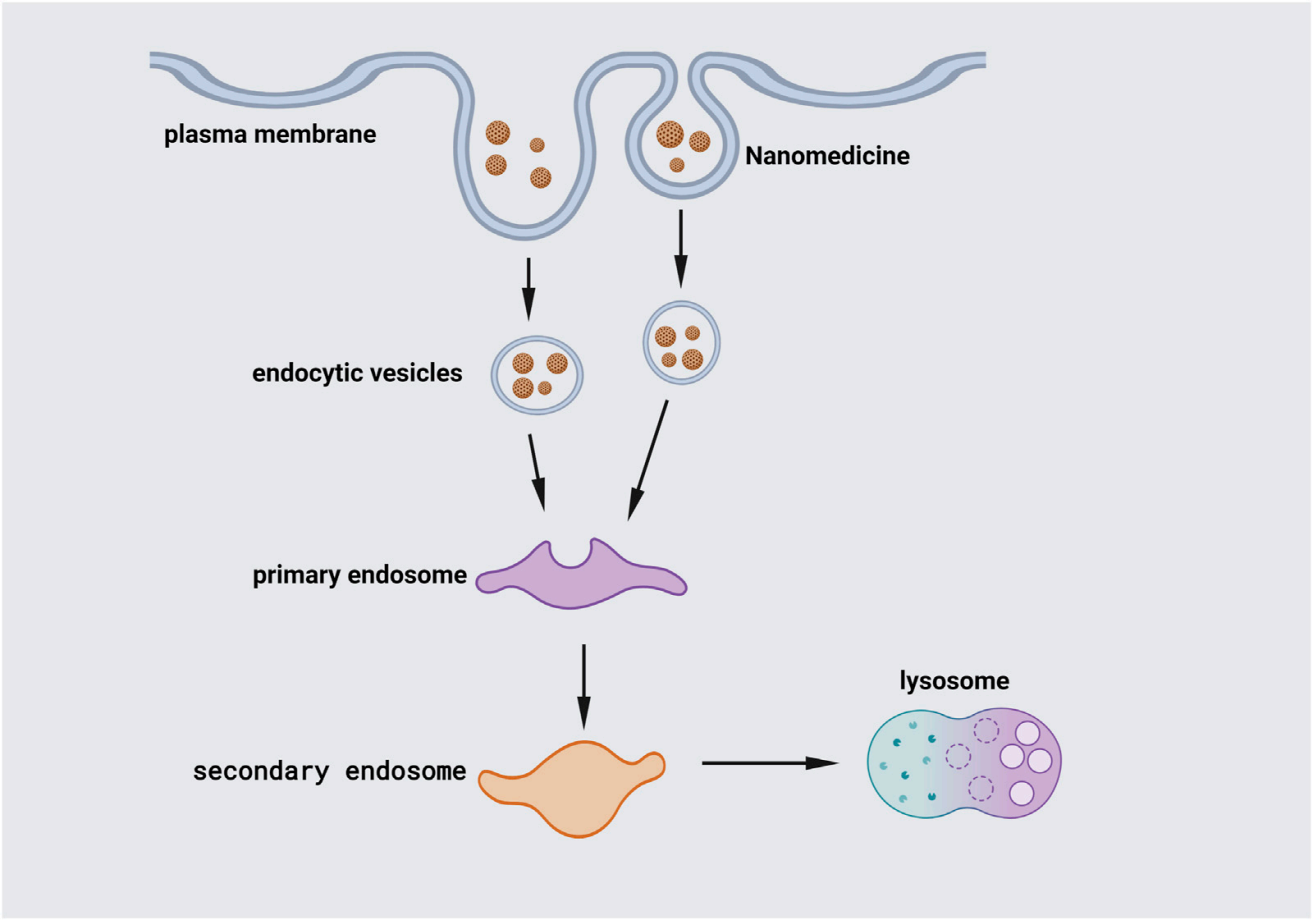
Internalization of NPs. Endocytosis, the major channel for passing the cellular membrane, allows NPs to enter the cell. Larger particles can be taken up by phagocytosis. The cell membrane engulfs the NPs, trapping them in the cellular vesicle. The vesicles are then uncoated and sent to intracellular components with specific functions. Early endosomes join vesicles together and shuttle particles to various locations. After that, the early endosome grows into the late endosome, which fuses with lysosomes.

The chosen endocytosis pathway determines the fate of NPs in cells. Internalization of NPs into cells is influenced by their morphology, which determines their behavior and biological function *in vivo* (Figure 4). However, several studies have shown that the physical features of NPs, such as size, charge, shape, distinct endocytic types of machinery in different cell types, aggregation state, and surface chemistry, might impact the transport pathway (Kou et al., 2013; Rascol et al., 2017).

#### 5.1.1 Size of NPs

The size of NPs has a significant impact on their cellular absorption. The nanomedicines must accumulate in the target tissues to be efficacious, and the NPs must be smaller than the mean pore size of the target tissue vasculature. In research, the size and shape of NPs are also of great significance in resolving pharmacokinetics and biodistribution issues. The appropriate size prevents the rapid excretion of NPs from the body, so as to make it work more effectively at the disease sites (Jurj et al., 2017).

Endocytosis is the mechanism by which cells take up biomolecules. Studies have shown that NPs with appropriate size can enter cells via the same method, and various types of particles may use different endocytic processes depending on their size (Pan et al., 2007; Zhu et al., 2013). Larger solid particles enter cells more efficiently via phagocytosis. For instance, it is reported that larger gold NPs can be localized in the nucleus of HeLa cells during cell division (Beaudet et al., 2017). Smaller particles (< 100 nm) adhere to Clathrin and Caveolae proteins and form endocytosis, helping cells phagocytize particles (Kashani et al., 2018). Besides, several investigations have shown that the optimal size for efficient cellular uptake of gold NPs is 50 nm (Jiang et al., 2008; Albanese and Chan, 2011; Huang et al., 2012). The results also show that internalization efficiency on human adipose-derived stem cells of spherical NPs in the range of 30–50 nm is higher than that of NPs with sizes of 15, 75, and 100 nm (Sohrabi Kashani and Packirisamy, 2021).

#### 5.1.2 Shape of NPs

Compared with non-spherical counterparts, spherical NPs have a higher level of internalization (Sohrabi Kashani et al., 2020). Even though nanospheres are promising candidates for drug delivery, anisotropic nanostructures may be more efficient due to larger surface/volume ratios and the ability to deliver higher drug concentrations to the desired regions (Sun et al., 2014). Because of the various contact surfaces between NPs and the cell membrane, NPs with different shapes have different ability to penetrate cells. A typical example is Nanorods, which have poorer internalization ability than spherical particles and require more time to wrap around the membrane (Chithrani et al., 2006). The ability of human breast cancer cells to absorb 14 and 74 nm spherical gold NPs is 5-fold and 3.75-fold higher, respectively, than that of 74 × 14 (nm) rod-shaped gold NPs (Chithrani et al., 2006). Furthermore, researchers have discovered that triangular NPs enter cells more efficiently than other forms, while gold nanostars have the lowest ability. They have also discovered that each shape of NP permeates the cell membrane using different endocytosis pathways, emphasizing the importance of NP geometry in determining intracellular fate (Xie et al., 2017).

**FIGURE 5:**
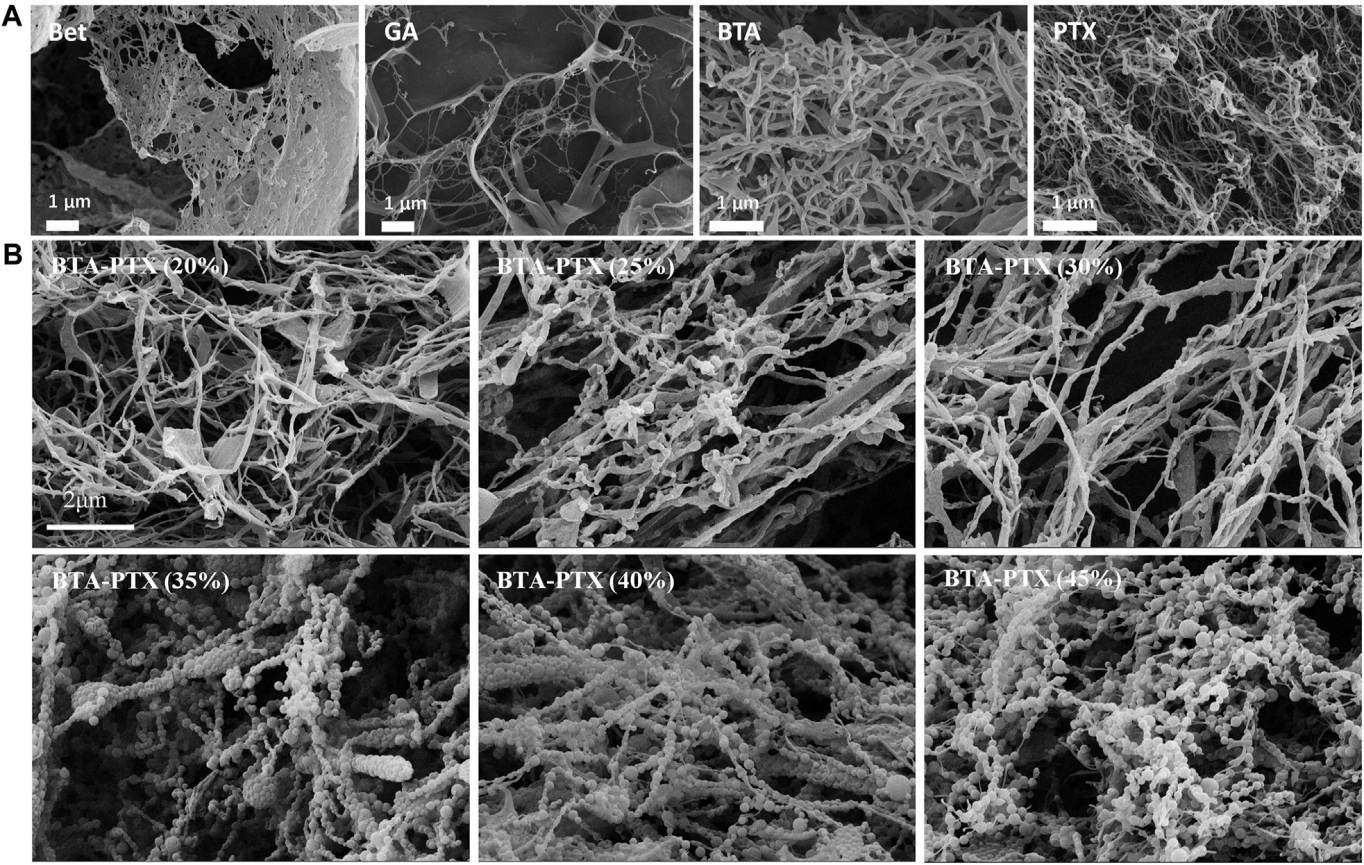
SEM images of NPs (Wang et al., 2020b). **(A)** SEM images of BTA, GA, BTA and PTX self-assemblies NPs. Self-assembly morphologies of GA, BTA, Bet and PTX are nanofibers with particle diameters greater than 600 nm. **(B)** Effect of BTA and PTX interaction at different ratios on the morphology of NPs. As the increase of PTX, the hybrid NPs gradually changes from nanofibers to nanospheres. When the mass ratio of PTX is greater than 35%, the spherical morphology gradually decreases and gradually transforms into nanofibers. Abbreviations: Bet: botulin; GA: glycyrrhetinic acid; BTA: Betulonic acid; PTX: paclitaxel; BTA-PTX: Co-assembled NPs formed by the interaction of BTA and PTX (different mass percentages).

To study the morphology of NPs, various characterization techniques have been developed, among which the most relevant are microscopic techniques including polarized light microscopy, scanning electron microscopy (SEM), and transmission electron microscopy (TEM) (Crouzier et al., 2021). The principle of electron scanning underpins the SEM technique, which delivers all available information about NPs at the nanoscale level. Utilizing this technique, people can analyze not only the shape of nanomaterials, but also the dispersion of NPs in the bulk or matrix (Saeed and Khan, 2014; Saeed and Khan, 2016). Nevertheless, TEM is based on the principle of electron transmittance, and it may provide information about the bulk material from low to high magnification. The quadrupolar hollow shell structure of Co_3_O_4_ NPs observed by TEM is an example of TEM providing vital information on two or more layered materials (Wang et al., 2013), and it can investigate the interactions and morphological structure of various nanomaterials (Figure 5) (Wang et al., 2020b).

### 5.2 Biosafety of NPs

Although NPs offer a speedier and more effective treatment for diseases like cancer and inflammation, their biosafety must be considered. It is suggested that researchers should closely limit cellular absorption and monitor the distribution and therapeutic efficacies of NPs to reduce the cytotoxicity of NPs and produce a safe and effective drug delivery system.

The size of NPs has been proven to alter their cytotoxicity in studies. According to certain research, gold NPs with smaller diameters are more hazardous to cells than NPs with larger diameters (Pan et al., 2007). This is because smaller NPs have a larger surface area to volume ratio, thereby interacting with cellular and subcellular compartments more efficiently. Smaller particles, furthermore, are more likely to reach intracellular regions like the mitochondria and nucleus, making them more hazardous (Kumar et al., 2017). In sum, the size of NPs is not only critical for targeting specific cellular localization, but also influences cell cytotoxicity (Lundqvist et al., 2011; Li and Lee, 2020). Furthermore, researchers have raised several safety issues that must be addressed, such as the cytotoxicity of therapeutic NPs when combined with various types of compounds, implying that the selection of nanoparticle material is critical (Jurj et al., 2017). The respiratory system is a possible site of NP toxicity action because it is the direct entry location for breathed particulate matter (Ferreira et al., 2013). NPs tend to aggregate in hard water and seawater and are highly influenced by the precise type of organic matter or other natural particles (colloids) in freshwater, but this has not to be extensively explored as part of ecotoxicological investigations yet (Handy et al., 2008).

### 5.3 Nanobiointeraction

Cellular mechanobiological features, including elasticity and adhesion, are critical in the advancement of cell function and tumor research. Understanding nanobiological interactions (NBI) will aid the development of nanoparticle technologies for controlling cancer progression by modifying cellular dynamics. Researchers have monitored the possible mechanobiological response of recipient cells as a standard method to assess the effectiveness of nanomedicines. The uptake of NPs by cells and their direct or indirect interactions with intracellular compartments have the potential to disorganize cytoskeletal structures, altering cell dynamics and functions (Wang et al., 2019). As a result, mechanobiological measurements are recommended for studying nanobiointeractions and determining how NPs affect cell mechanobiological features.

NBI behavior can be evaluated from a mechanical point of view. The presence of NPs within cells may have an impact on cell function directly or indirectly which may be reflected in cell mechanics or mechanobiology. Mechanobiology is a new multidisciplinary field integrating biology, bioengineering, and biophysics. To be specific, it studies the influence of cell mechanics and mechanical forces on cell activities, cell morphogenesis, and illnesses like cancer (Krieg et al., 2019). Mechanical characteristics of cells play an important role in perception and response to their external environment in mechanobiology (Kim et al., 2009; Kashani and Packirisamy, 2020; Narasimhan et al., 2020). The methods for studying NBI are atomic force microscopy (Li et al., 2017), micropipette aspiration (Trickey et al., 2000), optical tweezers (Bellini et al., 2010), and magnetic twist cytometry (Coughlin et al., 2006). In spite of several steps and long processing time, these approaches can offer high-resolution measurements of single-cell mechanobiology. In addition, mechanobiological characteristics can be measured at a faster rate with micro-electromechanical systems and microfluidic devices (Gossett et al., 2012; Byun et al., 2013).

## 6 Animal Husbandry Applications of NPs

Nanotechnology has facilitated the treatment of animal diseases in therapeutics, diagnostics, tissue engineering, vaccine production, and other areas. Nanomaterials and nanotechnology have already been used in animal health and production, animal breeding and reproduction, and animal nutrition.

### 6.1 Nanotechnology in Animal Nutrition and Health

Nanominerals and nanoemulsion technologies offer several benefits for the manufacture and use of cattle and poultry feed, including lower costs, fewer additives, and growth-promoting and immune-modulating properties (El-Sayed and Kamel, 2020). Nanominerals can also inhibit harmful pathogens in feed, regulate the process of rumen fermentation, and address reproductive problems in cattle and sheep herds (Laurent et al., 2008). Nanominerals have also been used to treat animal diseases. Nanozinc oxide, for example, can increase the development rate, immunity, and reproductive performance of farm animals and birds, as well as lower the incidence of diarrhea in piglets (Hassan et al., 2021). Nanozinc has been found to boost milk yield and lower the number of somatic cells in dairy cows with recessive mastitis.

In animal medicine, emerging nanomedicines have several advantages over traditional treatments, one of which is their self-control ability. For instance, when a peptide chain is combined with gentamicin, gentamicin medicine can be rendered inactive as long as the connecting chain is intact. Only proteases produced by *Pseudomonas aeruginosa* can break down the linker chain, hence gentamicin is only released and activated in the presence of *P. aeruginosa* (Suzuki et al., 1998; Soppimath et al., 2002). When it comes to the use of veterinary pharmaceuticals in livestock farms, nanomaterials can transport drugs directly to target cells, reduce drug dosage, drug leftovers and withdrawal time in pasture animals.

Liquid vitamins prepared by nanotechnology can be used in poultry feed. This nanoscale vitamin is meant to transfer vitamins and other nutrients straight into the bloodstream via the gastrointestinal tract, boosting bioavailability (Shabani et al., 2019). NPs can also reduce the need for preservatives and eliminate feed odors that are irritating to animals (Reddy et al., 2020). They can also increase nutrient dispersibility and feed durability. Feed ingredients can be microencapsulated to protect them from light and oxidation, to prevent them from being degraded by proteases and other digestive enzymes, and to keep them stable at various pH levels and temperatures. They have superior dispersibility in use, allowing for better mixing of fat-soluble additives in the feed, and extending their service life during storage. Nanotechnology has promise in animal medicine and production, but it needs to be tested further in industrial practice before it can be fully developed and utilized.

### 6.2 Animal Reproduction and Nanotechnology

Nanotechnology has been widely applied in animal breeding and reproduction, including the diagnosis and treatment of animal reproductive problems, the detection of estrus, and the isolation and freezing of sperm. The use of nanodevices during calving directly affects the mother and addresses reproductive issues such as placental retention, which has been applied in several stages of production (Swain et al., 2015). For the diagnosis of animal reproductive tract infectious diseases and hormonal disorders, as well as the detection of estrus, researchers have developed a nanosensor with a nanosized and highly sensitive biomolecular probe (Saragusty and Arav, 2011). Nanotechnology can also be utilized to separate sperm and oocytes, as well as transport nanocapsules containing bull sperm to cows for direct artificial insemination at high temperature. Biochips and nanomaterials have also been utilized to determine the gender of a fetus (Joanitti and Silva, 2014). Some metal NPs, such as cadmium, are poisonous at low concentrations, and researchers are working on producing NPs as animal sterilization contraceptives. NPs also provide sustained release of reproductive hormones in animals, preventing inactivation and degradation of certain hormones and vitamins due to oxidation (e.g., vitamins and steroid hormones) or hydrolysis (e.g., gonadotropins) (Joanitti and Silva, 2014). The effects of ultrafast freezing and rapid and uniform thawing of animal sperm can be obtained by microinjection of propylene glycol, a cryoprotectant containing metal NPs. Nanotechnology can also be used for cryopreservation of sperm, oocytes, or embryos (Saragusty and Arav, 2011).

In summary, nanotechnology plays a pivotal role in animal husbandry, having positive impacts on farmed animals in many aspects. Hence, it will be valuable to do in-depth research. However, the research of pasture nanotechnology is relatively simple and only plays a role in animal feeding and production these days. Therefore, more roles need to be investigated, such as nutritional control mechanisms and anti-disease effects.

## 7 Conclusion and Prospect

In the future, it is possible to use more types of nanomaterials and related nanoformulations in animal husbandry in the wake of the progress in nanotechnology in biomedicine. Advances in nanotechnology will accelerate the development of medical technology, resulting in changes in treatment methods of humans and animals. As a multidisciplinary field, research and development of nanotechnology include biology, chemistry, engineering, medicine, and physics. As a result, many features of NBI must be considered while designing safe and effective NP-based therapeutic and diagnostic systems.

Currently, there are many problems to be resolved. For example, it is unclear how physicochemical properties of NPs influence mechanobiology, to what extent NPs can change mechanobiological capabilities of cancer cells, and most critically, how these changes affect the actual processes. In addition, more research is needed to determine if NPs play a beneficial or detrimental function in anti-inflammatory and anti-tumor progression. NPs currently face problems such as excessive cost, insufficient large-scale production capacity, safety issues, and ineffective oversight, all of which impede clinical translation. However, with rapid advancements in nanomaterial technology and nanodrug delivery systems, these challenges are expected to be overcome, paving the way for successful clinical applications.

Although NPs have benefits in animal genetic breeding and illness therapy, the mechanisms of action have not been elucidated yet. Nanotechnology application in animal husbandry and veterinary care is not only represented in the prevention and control of animal diseases, but also in animal nutrition, reproduction, and animal welfare. This provides the breeding business with better management systems and breeding models. To sum up, nanotechnology opens a world of possibilities for animal medicine and health research, as well as revolutionary solutions to traditional veterinary problems.
